# The High Level of Tertiary Lymphoid Structure Is Correlated With Superior Survival in Patients With Advanced Gastric Cancer

**DOI:** 10.3389/fonc.2020.00980

**Published:** 2020-07-07

**Authors:** Wenting He, Dachuan Zhang, Hong Liu, Tongbing Chen, Jun Xie, Lei Peng, Xiao Zheng, Bin Xu, Qing Li, Jingting Jiang

**Affiliations:** ^1^Department of Oncology, The Third Affiliated Hospital of Soochow University, Changzhou, China; ^2^Department of Pathology, The Third Affiliated Hospital of Soochow University, Changzhou, China; ^3^Department of Tumor Biological Treatment, The Third Affiliated Hospital of Soochow University, Changzhou, China; ^4^Jiangsu Engineering Research Center for Tumor Immunotherapy, Changzhou, China; ^5^Institute of Cell Therapy, Soochow University, Changzhou, China

**Keywords:** tertiary lymphoid structure, gastric cancer, prognosis, MECA-79, tumor microenvironment

## Abstract

**Background:** A tertiary lymphoid structure (TLS) is a crucial component of the tumor microenvironment, which reflects the anti-tumor immune response in the host. The aim of the present study was to carry out a histopathological evaluation for TLS and assess its prognostic value in gastric cancer (GC).

**Methods:** A total of 1,033 cases that have received a gastrectomy were reviewed, including 914 in the primary cohort and 119 in the validation cohort. TLS was assessed by optical microscopy and verified by immunohistochemistry. A total of five histopathological evaluation methods were compared in the primary cohort and validated in the validation cohort. In addition, MECA-79 and CD21 were used to verify the accuracy of the histopathological scoring system for TLS. The association among TLS, clinicopathological parameters, and patient prognosis was analyzed.

**Results:** TLS as assessed by morphology and immunohistochemistry were significantly correlated and consistent. The morphological evaluation of TLS was accurate. Typically, the high level of TLS was significantly correlated with tumor size (*P* = 0.047), histological grade (*P* = 0.039), pTN stage (*P* = 0.044), and WHO subtype (*P* < 0.001). In addition, TLS^hi^ was a positive indicator of overall survival, as determined by Kaplan–Meier survival (*P* = 0.038) and multivariate Cox regression analyses (hazard ratio = 0.794, 95% CI: 0.668–0.942, *P* = 0.008). According to the results, TLS^hi^ had a positive effect on the primary cohort patients with pTN stages II and III (*P* = 0.027, *P* = 0.042).

**Conclusions:** The histopathological evaluation of TLS was accurate. Diagnosis based solely on hematoxylin and eosin staining of the sections did not easily distinguish tumor-associated TLS. The density of TLS in the center of the tumor was found to be more indicative of patient prognosis than TLS in the invasive margin, with the levels of total TLS shown to best correlate with overall survival in patients with advanced-stage GC.

## Introduction

The tumor microenvironment (TME) is the internal environment where a tumor develops and where a functional complex of interactions among tumor cells, stromal cells, and tumor-infiltrating lymphocytes (TIL) takes place. The TME status reflects the interaction between the host immune response and tumor progression. Typically, TIL is a key component in TME that reflects the anti-tumor immune response in the host. It is also crucial for suppressing cancer progression and has implications for the success of the host anti-tumor immune response against certain solid tumors (1–3). A tertiary lymphoid structure (TLS), an organ-like structure, recruits naive T and B cells into the tumor site via chemokines or cytokines. TLS is similar to a secondary lymphoid structure (SLO) in terms of structure and function (4–7). Despite the known role of TLS in tumor progression, its formation and function in solid tumors have not been thoroughly explained so far. In recent studies, TLS has been detected in primary (8) and metastatic tumors (7), and the high density of TLS has been found to be positively correlated with the prognosis for colorectal (9), lung (10), breast cancer (11), and malignant melanoma (12). However, the mechanisms that promote an anti-tumor immune response, as well as the association between TLS and patient prognosis, in gastric cancer (GC) remain unclear (13). High endothelial venules (HEVs) are the characteristic vessels of SLO and TLS. Of note, MECA-79 (14, 15) expressed on the HEVs binds to CD62L, and its expression on lymphocytes causes the latter to roll along HEVs and infiltrate the TME (12). Based on the results from the aforementioned studies, HEVs are the crucial sites for lymphocyte recruitment and TLS accounts for an important source of TIL. The aim of the present study was to assess the distribution and the prognostic value of TLS in GC by reviewing a large number of cases. In addition, immunohistochemically stained MECA-79 and CD21 were used to verify the accuracy of the morphological TLS scoring system. The association among TLS, clinicopathological parameters, and patient prognosis was analyzed.

## Materials and Methods

### Patient and Tissue Samples

In this retrospective cohort study, data were collected from the Department of Pathology of the Third Affiliated Hospital of Soochow University between 2002 and 2008. The patient inclusion criteria were as follows: (i) patients pathologically diagnosed with primary gastric adenocarcinoma, (ii) patients who were naïve to preoperative chemotherapy or radiotherapy, (iii) patients with adequate formalin-fixed and paraffin-embedded (FFPE) tissue blocks, (iv) patients with at least one slide containing the tumor invasive margin (IM), and (v) patients with complete medical records and follow-up information. Finally, a total of 1,033 cases were included in this study. To construct and validate the analyses for TLS, 914 patients were enrolled into the primary cohort between 2004 and 2008, while the remaining patients (119 cases) were included as the external validation cohort between 2002 and 2003. The Tumor Pathological Staging System (8th Edition) of the Union for International Cancer Control/American Joint Committee on Cancer was adopted for GC classification. The patient survival intervals were available and dated to the end of January 2018. The study protocol was approved by the Ethics Committee of Soochow University and conducted in accordance with the Declaration of Helsinki.

### Histopathological Evaluation of Sections Using Hematoxylin and Eosin Staining

The aim of the present study was to evaluate the methodology for a TLS scoring system in GC. TLS evaluation was retrospectively performed using H&E staining. In brief, the tumor tissue sections were reviewed independently by four pathologists who were trained on the TLS scoring system and blinded to the clinical data. During the subsequent scoring process, any problematic case was discussed jointly by two or more of the aforementioned pathologists. No uniform standard was available for the histopathological TLS scoring system, and TLS was defined as a cumulative area of ectopic lymphocytes (>200-fold microscopic field; 1 mm in diameter). According to the criterion of previous studies (16–18), the tumor area was subdivided in the center of the tumor (CT) and the IM. Next, all available complete sections were morphologically analyzed for the (i) number of TLS and (ii) percentage of the CT or the IM region occupied by TLS in 10% increments (5 and 95% were allowed at extremes) (19). Subsequently, TLS evaluation was conducted in both the CT and the IM regions separately, and the number and the percentage were then incorporated. Following the TLS assessment, a set of TLS scores was obtained, including (i) TLS-CT number (TLS-CT-N), which indicated the number of TLS in the CT region, (ii) TLS-CT density (TLS-CT-D), which was calculated by multiplying a score of 1 with the percentage of TLS in the CT region and reflected the distribution and the density of TLS in the CT region, (iii) TLS-IM number (TLS-IM-N), which represented the number of TLS in the IM region, (iv) TLS-IM density (TLS-IM-D), which was calculated by multiplying a score of 3 with the percentage of TLS in the IM region and reflected the distribution and the density of TLS in the IM region, and (v) TLS-SUM, which indicated the TLS total score in both the CT and the IM regions (namely, the sum of TLS-CT-D and TLS-IM-D).

### The Repeatability and the Accuracy of TLS Evaluation

The repeatability and the accuracy of the TLS evaluation were double-checked. First, the methodology of the TLS evaluation was obtained in the primary cohort, and the TLS evaluation was validated in the validation cohort. Secondly, immunohistochemically stained MECA-79 and CD21 were used to verify the accuracy of the morphological evaluation.

A total of 63 randomly selected cases in the primary cohort were immunologically stained for MECA-79 (1:125 dilution, RRID: AB_493556, BioLegend). HEVs, the special venules in TLS, were selected to verify the accuracy of the TLS scoring system. MECA-79 recognized the sulfate-dependent carbohydrate epitopes expressed on the endothelial cells of HEVs. Meanwhile, 119 cases in the validation cohort were immunologically stained for CD21 (ready-to-use, Cat# RMA-0811, MXB Biotechnologies). The follicular dendritic cells (FDC) marked by CD21 were regarded as a representative lymphoid follicle in TLS. The FFPE tumor tissues were cut into 4-μm-thick sections, dewaxed in xylene, rehydrated, and exposed to gradient ethanol solutions. Cases with a positive immunohistochemical staining for MECA-79 and CD21 in the cytoplasm were considered as positive cases. The number of MECA-79^+^ vessels and CD21^+^ lymphoid follicles was recorded.

### Statistical Analysis

IBM SPSS statistical software v24.0 (IBM Corp) and GraphPad Prism 6 (GraphPad Software) were used for the statistical analysis. Correlation analysis, χ^2^ test, consistency test, Kaplan–Meier survival analysis, and Cox regression analysis were used as appropriate. All statistical analyses were two-sided, and *P* < 0.05 was considered to indicate a statistically significant difference.

## Results

### Prognostic Value of TLS in GC

**Figure 1:**
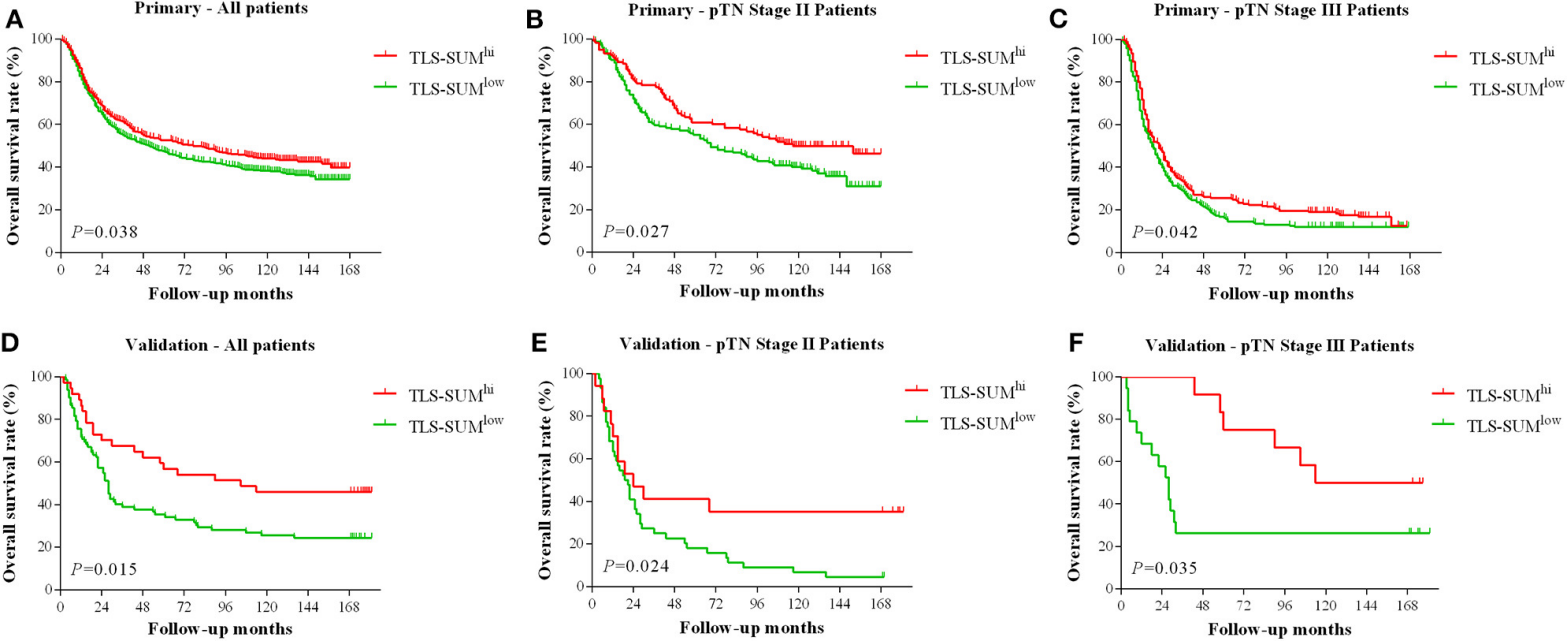
The hierarchical Kaplan–Meier survival curves for TLS-SUM in GC. TLS-SUM^hi^ is associated with better prognosis in GC patients from the primary cohort **(A)** and the validation cohort **(D)**. The contribution of TLS to the prognostic power of pTN stages II and III patients was evaluated from the primary cohort **(B,C)** and the validation cohort **(E,F)**. TLS, tertiary lymphoid structure; GC, gastric cancer.

The analysis was conducted based on data from the primary cohort. To make the scoring system simpler for statistical analysis, the variables were transformed into binary variables according to the median (Supplemental Table I). As shown by the Kaplan–Meier survival analysis, high levels of TLS-CT-D or TLS-SUM were correlated with superior survival in patients (χ^2^ = 4.013, *P* = 0.045; χ^2^ = 4.298, *P* = 0.038, Table 1, Figure 1A). In addition, the contribution of TLS to the prognostic power of each pTN stage was evaluated (Table 1). The results suggested that TLS-SUM had a protective effect on patients with pTN stages II and III (*P* = 0.027, *P* = 0.042, Table 1, Figures 1B,C). To verify the statistical analysis results, an external validation cohort was used to test the variables. The prognostic value of TLS-SUM in the validation cohort was the same as that in the primary cohort (*P* = 0.024, *P* = 0.035, Table 1, Figures 1D–F). High levels of TLS-IM-N were correlated with better survival in pTN stage II patients from the primary and the validation cohorts (*P* = 0.006, *P* = 0.034, Table 1, Supplemental Figures 1A,B). A total of 325 cases with different levels of TLS-CT-D and TLS-IM-N were grouped: 169 TLS-CT-D^high^ TLS-IM-N^low^ cases and 156 TLS-CT-D^low^ TLS-IM-N^high^ ones. The TLS-CT-D^high^ TLS-IM-N^low^ was correlated with better survival in all patients and pTN stage I patients from the primary cohort (*P* = 0.031, *P* = 0.032, Supplemental Figures 1C,D).

**Table 1:** Hierarchical K–M survival analysis of tertiary lymphoid structure (TLS) and pathological tumor and lymph node (pTN).

**Hierarchical definition**		**TLS in primary (high/low)**		**χ^**2**^**	***P* value**	**TLS in validation (high/low)**		**χ^**2**^**	***P* value**
**TLS score**	**pTN stage**	**Number**	**Mean overall survival (OS)**			**Number**	**Mean OS**		
TLS-CT-N	I	133/103	148.6/144.0	0.547	0.460	14/13	138.6/130.3	0.215	0.643
	II	114/160	103.6/89.0	3.071	0.080	11/20	97.0/81.5	0.213	0.645
	III	223/181	48.3/39.5	2.912	0.088	24/37	57.9/40.1	1.701	0.192
	I–III	470/444	90.1/81.7	3.326	0.068	49/70	90.3/68.8	2.632	0.105
TLS-CT-D	I	139/97	148.4/144	0.650	0.420	12/15	141.7/129.1	0.454	0.500
	II	119/155	103.4/88.4	3.278	0.070	11/20	97.0/81.5	0.213	0.645
	III	225/179	47.7/40.2	2.220	0.136	25/36	67.7/32.4	5.261	**0.022**
	I–III	483/431	90.3/81.0	4.013	**0.045**	48/71	93.4/66.9	3.401	0.065
TLS-IM-N	I	104/132	139.1/152.4	3.323	0.068	7/20	114.7/141.3	0.447	0.504
	II	137/137	105.8/84.1	7.552	**0.006**	13/18	121.2/61.6	4.519	**0.034**
	III	229/175	45.7/42.5	0.977	0.323	18/43	74.9/35.3	5.417	**0.020**
	I–III	470/444	84.0/87.9	0.555	0.456	38/81	98.7/67.3	4.825	**0.028**
TLS-IM-D	I	106/130	139.6/152.1	2.826	0.093	7/20	114.7/141.3	0.447	0.504
	II	138/136	105.4/84.3	7.160	**0.007**	14/17	114.9/63.3	3.760	0.053
	III	230/174	45.5/42.6	0.848	0.357	18/43	74.9/35.3	5.417	**0.020**
	I–III	474/440	84.1/87.8	0.460	0.498	39/80	97/67.8	4.487	**0.034**
TLS-SUM	I	127/109	145.3/148.0	0.001	0.969	8/19	122.5/140.0	0.191	0.662
	II	120/154	105.1/87.0	4.866	**0.027**	12/19	127.7/60.8	5.126	**0.024**
	III	210/194	49.3/38.7	4.153	**0.042**	17/44	76.2/35.5	4.424	**0.035**
	I–III	457/457	90.7/81.1	4.298	**0.038**	37/82	103.7/65.9	5.928	**0.015**

*Values in bold signify p < 0.05*.

Cox regression analysis was performed to evaluate the prognostic significance of TLS and the clinicopathological parameters [including age, tumor size, lymph node (LN) metastasis, vessel invasion, histopathological grade, pTN stage, WHO subtypes, and sets of TLS]. In the univariate Cox regression analysis, TLS-CT-D, TLS-SUM, and all the clinicopathological parameters were found to be independent prognostic factors for overall survival. Moreover, the LN metastasis information was contained in the pTN stage parameter and excluded from the multivariate Cox regression model. The multivariate Cox regression analysis was performed based on the TLS-CT-D and the TLS-SUM data [hazard ratio (HR) = 0.833, 95% CI: 0.702–0.989, *P* = 0.037; HR = 0.794, 95% CI: 0.668–0.942, *P* = 0.008, Supplemental Table II]. Of note is that TLS had a HR score of <1, suggesting that the high level of TLS showed a protective effect on patient survival. To verify the statistical analysis, Cox regression analysis was performed in the validation cohort. In univariate and multivariate Cox regression analyses, TLS-SUM was identified as the independent prognostic factor (HR = 0.496, 95% CI: 0.252–0.973, *P* = 0.041; HR = 0.411, 95% CI: 0.229–0.740, *P* = 0.003, Table 2).

**Table 2:** Univariate and multivariate Cox regression analyses of tertiary lymphoid structure (TLS)-SUM and clinicopathological parameters.

**Clinicopathological parameters**	**Multivariate analysis in primary**			**Multivariate analysis in validation**		
	**HR**	**95% CI**	***P* value**	**HR**	**95% CI**	***P* value**
Age (≤ 50/>50)	1.954	1.472–2.594	**<0.001**	1.924	1.010–3.667	**0.047**
Tumor size (≤ 5 cm/>5 cm)	1.101	0.917–1.321	0.303	1.272	0.740–2.189	0.384
Vessel invasion (-/+)	1.209	1.013–1.443	**0.035**	1.316	0.648–2.670	0.448
Histological grade						
Well		Reference			Reference	
Moderately	0.862	0.414–1.794	0.691	4.122	0.506–33.566	0.186
Poor	1.094	0.520–2.301	0.812	4.525	0.539–37.978	0.164
pTN (I–III)						
I		Reference			Reference	
II	3.295	2.290–4.742	**<0.001**	2.343	0.988–5.558	0.053
III	8.334	5.788–12.000	**<0.001**	3.246	1.367–7.705	**0.008**
WHO subtypes						
Tubular		Reference			Reference	
Mucinous	0.780	0.579–1.052	0.103	0.636	0.300–1.350	0.239
Papillary	1.311	0.899–1.913	0.160	0.739	0.292–1.872	0.523
Poorly cohesive	1.006	0.795–1.273	0.962	0.903	0.382–2.137	0.816
Undifferentiated	1.182	0.861–1.624	0.301	2.207	1.000–4.873	0.050
TLS-SUM (low/high)	0.794	0.668–0.942	**0.008**	0.411	0.229–0.740	**0.003**

*Values in bold signify p < 0.05*.

### Association Between TLS and Clinicopathological Parameters

Details of the clinicopathological parameters, TLS-SUM, and TLS-CT-D are presented in Table 3. TLS was significantly correlated with tumor size, histological grade, pTN stage, and WHO subtype (*P* < 0.05, Table 2). These data revealed that the high levels of TLS were associated with clinicopathological parameters, such as smaller tumor size and earlier pTN stage, and indicated better prognosis. TLS might serve as a potent prognostic indicator, which can be as crucial as a clinicopathological parameter.

**Table 3:** Relationship between tertiary lymphoid structure (TLS) and clinicopathological parameters.

**Clinicopathological parameters**	**TLS-SUM**		***χ*^2^**	***P* value**	**TLS-CT-D**		***χ*^2^**	***P* value**	**TLS-IM-N**		***χ*^2^**	***P* value**
	**High**	**Low**			**High**	**Low**			**High**	**Low**		
Age (year)												
>50	387	403	2.389	0.122	410	380	2.091	0.148	405	385	0.057	0.811
≤ 50	70	54			73	51			65	59		
Tumor size (cm)												
>5	220	250	3.942	**0.047**	225	245	9.599	**0.002**	250	220	1.212	0.271
≤ 5	237	207			258	186			220	224		
Lymph node metastasis											
Yes	271	292	2.040	0.153	290	273	1.048	0.306	299	264	1.668	0.196
No	186	165			193	158			171	180		
Vessel invasion												
Yes	157	158	0.005	0.945	155	160	2.553	0.110	169	146	0.956	0.328
No	300	299			328	271			301	298		
Histological grade												
Well	22	20	6.512	**0.039**	26	16	7.396	**0.025**	17	25	2.795	0.247
Moderately	146	183			155	174			165	164		
Poor	289	254			302	241			288	255		
pTN stage												
I	127	109	6.226	**0.044**	139	97	14.531	**0.001**	104	132	9.808	**0.007**
II	120	154			119	155			137	137		
III	210	194			225	179			229	175		
WHO subtypes												
Tubular	296	277	24.119	**<0.001**	309	264	15.867	**0.003**	304	269	7.781	0.100
Mucinous	37	48			47	38			40	45		
Papillary	9	39			13	35			16	32		
Poorly cohesive	84	62			85	61			78	68		
Undifferentiated	31	31			29	33			32	30		

*Values in bold signify p < 0.05*.

### Accuracy of Morphological Evaluation of TLS

According to the abovementioned analyses, TLS-SUM was screened as the representative of morphological TLS evaluation. Immunohistochemical staining of MECA-79 and CD21 was used to verify the accuracy of the morphological TLS evaluation.

**Figure 2:**
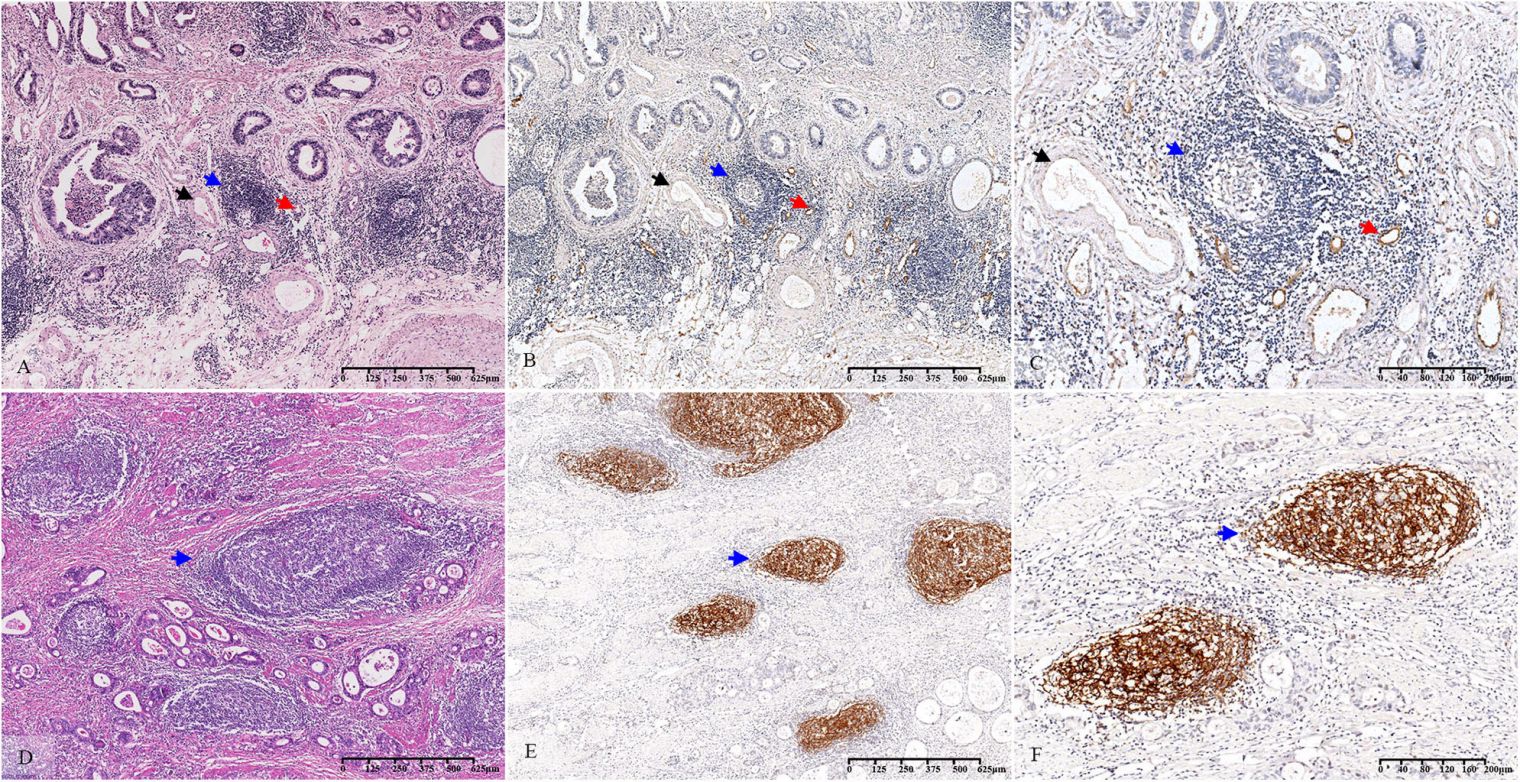
Histopathological observation and immunohistochemical verification of TLS. The TLS present in GC tissue **(A–D)** (blue arrow). MECA-79 and CD21 were used to verify the accuracy of the morphological evaluation of TLS. HEVs **(A–C)** (red arrow, MECA-79^+^) and venules **(A–C)** (black arrow, MECA-79^−^) are shown closing around the TLS. CD21-marked FDCs **(E,F)** (blue arrow, CD21^+^) are observed in the TLS. TLS, tertiary lymphoid structure; GC, gastric cancer; HEVs, high endothelial venules; FDCs, follicular dendritic cells.

Based on a statistical correlation analysis, the score of TLS-SUM was positively correlated with the number of MECA-79^+^ vessels (*r* = 0.696, *P* < 0.001, Supplemental Table III, Figures 2A–C). In a subsequent statistical analysis, all variables were transformed into binary variables. Thereafter, all 63 patients were divided into two groups according to the presence or the absence of MECA-79^+^ vessels, including 40 (63.5%) MECA-79^+^ cases and 23 (36.5%) MECA-79^−^ ones. According to the TLS-SUM, the 63 patients were subdivided into 44 high-level cases and 17 low-level cases. The results of the χ^2^ test and consistency analysis revealed a significant consistency agreement between MECA-79 and TLS-SUM (χ^2^ = 18.849, *P* < 0.001, κ = 0.565, *P* < 0.001, Supplemental Table III).

The CD21-marked FDC in the B cell follicles was also used to verify the accuracy of the morphological TLS evaluation. Upon analysis, the score of TLS-SUM was positively correlated with the number of CD21^+^ lymphoid follicles (*r* = 0.924, *P* < 0.001, Supplemental Table III, Figures 2D–F). The patients in the validation cohort were divided into two groups, according to the median: 51 CD21^hi^ cases and 68 CD21^low^ cases, with 37 cases having high levels of TLS-SUM and 82 low-level cases. Furthermore, the χ^2^ and consistency tests revealed a significant consistency between CD21 and TLS-SUM (χ^2^ = 71.593, *P* < 0.001, κ = 0.751, *P* < 0.001, Supplemental Table III).

## Discussion

TLS, an organ-like structure of the ectopic aggregated lymphocytes, was first identified in chronic inflammation and autoimmune diseases (5–7). In such pathological structures, tissues that harbor tumor antigens are infiltrated by cellular or molecular effectors of the immune system, which are then organized anatomically and functionally as those in SLO with B follicles and T-zone formation (9). TLS is a potential functional immune site for T lymphocyte activation, B cell maturation, and antibody production. According to recent studies, TLS has been detected in various tumors and found to be associated with favorable prognosis (7–12, 20–22). Nonetheless, there are no uniform histopathological diagnostic standards for TLS, and the prognostic value of TLS distribution remains unclear in the field of GC (4, 13). In the present study, a set of TLS scores was obtained by reviewing the H&E-stained sections. Based on the immunohistochemical detection of MECA-79 and CD21, the morphological TLS evaluation was accurate, which was verified by the external validation testing. By comparing the TLS between the CT and the IM regions, it was found that the presence of TLS in the CT region was correlated with better prognosis than that in the IM region, which was a novel finding. The gastric wall is anatomically classified into four layers: mucosa, submucosa, muscular layer, and serosa. The submucosa and the subserosa contain loose connective tissue as well as normal lymph nodes and ectopic lymphoid aggregates under homeostatic conditions as part of the digestion-associated lymphoid tissues, which play an essential role in the gastrointestinal immune response. The IM region located in the submucosa and the subserosa correspond to pTN stage I or III. In these two layers, the TLS may be associated with tumors or pre-exist, making them hard to distinguish using H&E-staining-based diagnosis. On the contrary, the muscular layer is composed of smooth muscle and barely contains lymphatic tissue in the normal gastric tissue. The IM region located in the muscular layer corresponding to pTN stage II and the TLS-IM were mainly associated with tumors in GC tissue. The results of the hierarchical Kaplan–Meier analysis confirmed this. High levels of TLS-IM were correlated with better survival in pTN stage II patients, but no correlation with survival was identified in pTN stages I–III patients. TLS-CT-D^high^ TLS-IM-N^low^ was correlated with better survival in all patients and in pTN stage I patients. Therefore, it is necessary that the ability to make this distinction be improved in future studies.

The formation and the function of TLS in solid tumors have not yet been thoroughly explained. At the cancer site, TLS may trigger a cancer-associated inflammation, and it is a crucial site for the production of antibodies that exert important effects on the polarization of macrophages and myeloid cells (23); this way, the derived suppressor cells promote cancer progression (24). Conversely, the antitumor immune response originating from TLS can potentially control tumor invasion and metastasis, which can thus enhance the efficiency and the specificity of T-cell priming and boost a faster T-cell reaction to tumor antigens *in situ* (9, 25). In addition, the present study also showed that TLS-SUM and TLS-CT-D were significantly correlated with tumor size, pTN stage, and histological grade. According to the above results, TLS^hi^ was associated with better prognosis for GC patients since TLS was a potential regulatory factor of tumor progression and metastasis. Malignant tumors were treated under the standard therapeutic guidelines based on TNM staging. In the present study, the prognostic power of TLS at different pTN stages was evaluated. As suggested by the Kaplan–Meier survival analysis results, TLS-SUM had a protective effect on patients at pTN stages II and III. Conversely, TLS-SUM expression made no statistically significant difference to patients at pTN stage I. We therefore speculated that the antitumor effect of TLS gradually increased during the early stage of tumor progression, which peaked at the middle and the late stages. This result was consistent with the three stages of tumor progression, namely, immune suppression, immune balance, and immune escape (26), leading us to believe that lymphocytes in TLS transformed during the tumor progression process and that the functional status of TLS also changed.

Based on univariate and multivariate Cox regression analyses, TLS-SUM was identified as an independent prognostic factor. These data showed that TLS-SUM served as a positive prognostic indicator that was as crucial as the clinicopathological parameters. Furthermore, patients with a high level of TLS-SUM had better prognosis than those with a low level of TLS, which was verified by the correlation between TLS and cancer mortality in the Kaplan–Meier survival analysis. In previous studies, the high density of TLS was found to be associated with good prognosis in tumor patients (9–12). This observation was also consistent with the present data in GC, which might interact with novel therapeutic approaches such as immunotherapy in GC (27–29). The morphological evaluation results showed clear TLS heterogeneity and prognostic value in GC. Consequently, TLS was predicted to represent a protective host immune system and an antitumor TME in GC. As a crucial factor and target of immunotherapy susceptibility, the lymphocyte components and their functional changes in TLS should be further examined.

## Data Availability Statement

All datasets generated for this study are included in the article/Supplementary Material.

## Ethics Statement

The studies involving human participants were reviewed and approved by The Ethics Committee of Soochow University. The patients/participants provided their written informed consent to participate in this study.

## Author Contributions

WH and DZ: conception and design of the study. DZ, TC, JX, and LP: collection and assembly of data. XZ, HL, and BX: statistical analysis of data. JJ and QL: study supervision. WH and DZ: drafting of the manuscript. All authors contributed to the article and approved the submitted version.

## Conflict of Interest

The authors declare that the research was conducted in the absence of any commercial or financial relationships that could be construed as a potential conflict of interest.

## Supplementary Material

Supplemental Figure 1 The Kaplan–Meier survival analysis of TL-IM-N at pTN stage II from the primary cohort **(A)** and the validation cohort **(B)**. According to the TLS-CT-D and the TLS-IM-N, the patients were divided into TLS-CT-D^high^ TLS-IM-N^low^ and TLS-CT-D^low^ TLS-IM-N^high^. The Kaplan–Meier survival curves for pTN stages I–III patients **(C)** and pTN stage I patients **(D)** from the primary cohort are shown.

Supplemental Table I Subgroup detail of histopathological evaluation of TLS.

Supplemental Table II Multivariate Cox Regression Analyses of TLS scores.

Supplemental Table III Relationship between TLS-SUM and MECA-79 for HEVs.

## Abbreviations

TIL: tumor infiltrating lymphocytes
TME: tumor microenvironment
TLS: tertiary lymphoid structure
GC: gastric cancer
CT: center of the tumor
IM: invasive margin
pTN: pathological tumor and lymph node
LN: lymph node
OS: overall survival
AUC: area under the ROC curve.
