# Supplementary material for: The High Level of Tertiary Lymphoid Structure Is Correlated With Superior Survival in Patients With Advanced Gastric Cancer
Source: Front Oncol. 2020 Jul 7;10:980. doi: 10.3389/fonc.2020.00980 (PMC7358602; doi:10.3389/fonc.2020.00980)
Supplement: Supplemental Table I — Subgroup detail of histopathological evaluation of TLS. [file Data_Sheet_1.docx]

Supplemental Table I. Subgroup detail of histopathological evaluation of TLS

| a set of | Primary | % | Validation | % | sub-group |
| --- | --- | --- | --- | --- | --- |
| TIL scoring | (*n*=914) |  | (*n*=119) |  | by median |
| Score 1: TLS-CT-N | |  |  |  |  |
| high | 470 | 51.42 | 49 | 41.18 | ≥2.667 |
| low | 444 | 48.58 | 70 | 58.82 | <2.667 |
| Score 2: TLS-CT-D | |  |  |  |  |
| high | 483 | 52.84 | 48 | 40.34 | ≥0.150 |
| low | 431 | 47.16 | 71 | 59.66 | <0.150 |
| Score 3: TLS-IM-N | | |  |  |  |
| high | 470 | 51.42 | 38 | 31.93 | ≥1.333 |
| low | 444 | 48.58 | 81 | 68.07 | <1.333 |
| Score 4: TLS-IM-D | | |  |  |  |
| high | 474 | 51.86 | 39 | 32.77 | ≥0.067 |
| low | 440 | 48.14 | 80 | 67.23 | <0.067 |
| Score 5: TLS-SUM | |  |  |  |  |
| high | 457 | 50.00 | 37 | 31.09 | ≥0.329 |
| low | 457 | 50.00 | 82 | 68.91 | <0.329 |

Supplemental Table II. Multivariate Cox Regression Analyses of TLS scores

| Clinicopathological | Multivariate analysis^a^ | | | Multivariate analysis^b^ | | |
| --- | --- | --- | --- | --- | --- | --- |
| parameters | *HR* | *95% CI* | *P* value | *HR* | *95% CI* | *P* value |
| Age (≤50/>50) | 1.980 | 1.492-2.627 | **<0.001** | 1.954 | 1.472-2.594 | **<0.001** |
| Tumor size (≤5 cm/>5 cm) | 1.089 | 0.908-1.308 | 0.358 | 1.101 | 0.917-1.321 | 0.303 |
| Vessel invasion (-/+) | 1.201 | 1.007-1.433 | **0.042** | 1.209 | 1.013-1.443 | **0.035** |
| Histological grade |  |  |  |  |  |  |
| Well |  | Reference |  |  | Reference |  |
| Moderately | 0.858 | 0.412-1.785 | 0.682 | 0.862 | 0.414-1.794 | 0.691 |
| Poor | 1.082 | 0.514-2.274 | 0.836 | 1.094 | 0.520-2.301 | 0.812 |
| pTN (I-III) |  |  |  |  |  |  |
| I |  | Reference |  |  | Reference |  |
| II | 3.258 | 2.263-4.692 | **<0.001** | 3.295 | 2.290-4.742 | **<0.001** |
| III | 8.339 | 5.790-12.008 | **<0.001** | 8.334 | 5.788-12.000 | **<0.001** |
| WHO subtypes |  |  |  |  |  |  |
| Tubular |  | Reference |  |  | Reference |  |
| Mucinous | 0.806 | 0.599-1.084 | 0.154 | 0.780 | 0.579-1.052 | 0.103 |
| Papillary | 1.336 | 0.916-1.948 | 0.132 | 1.311 | 0.899-1.913 | 0.160 |
| Poorly cohesive | 1.012 | 0.799-1.280 | 0.924 | 1.006 | 0.795-1.273 | 0.962 |
| Undifferentiated | 1.180 | 0.858-1.621 | 0.309 | 1.182 | 0.861-1.624 | 0.301 |
| TLS-CT-D (low/high) | 0.833 | 0.702-0.989 | **0.037** | - | - | **-** |
| TLS-SUM (low/high) | - | - | **-** | 0.794 | 0.668-0.942 | **0.008** |

Values in bold signify *p*<0.05

Multivariate analysis^a^ was based on the data of the TLS-CT-D.

Multivariate analysis^b^ was based on the data of the TLS-SUM.

Supplemental Table III. Relationship between TLS-SUM and MECA-79 for HEVs

| Immunohistochemistry | | Patients | TLS-SUM | | χ^2^ | *P* value | *R* | *P* value | *κ* | *P* value |
| --- | --- | --- | --- | --- | --- | --- | --- | --- | --- | --- |
|  |  | Num. | High | Low |  |  |  |  |  |  |
| MECA-79  (HEVs) | Positive | 40 | 37 | 3 | 18.849 | **<0.001** | 0.696 | **<0.001** | 0.565 | **<0.001** |
|  | Negative | 23 | 9 | 14 |  |  |  |  |  |  |
| CD21  (FDC) | High | 51 | 37 | 14 | 71.593 | **<0.001** | 0.924 | **<0.001** | 0.751 | **<0.001** |
|  | Low | 68 | 0 | 68 |  |  |  |  |  |  |

Values in bold signify *p*<0.05
